# Topical Administration of SLN-Based Gene Therapy for the Treatment of Corneal Inflammation by De Novo IL-10 Production

**DOI:** 10.3390/pharmaceutics12060584

**Published:** 2020-06-23

**Authors:** Mónica Vicente-Pascual, Itziar Gómez-Aguado, Julen Rodríguez-Castejón, Alicia Rodríguez-Gascón, Elisabetta Muntoni, Luigi Battaglia, Ana del Pozo-Rodríguez, María Ángeles Solinís Aspiazu

**Affiliations:** 1 Pharmacokinetic, Nanotechnology and Gene Therapy Group (PharmaNanoGene), Faculty of Pharmacy, Centro de investigación Lascaray ikergunea, University of the Basque Country UPV/EHU, Paseo de la Universidad 7, 01006 Vitoria-Gasteiz, Spain; monica.vicente@ehu.eus (M.V.-P.); itziar.gomez@ehu.eus (I.G.-A.); julen.rodriguez@ehu.eus (J.R.-C.); alicia.rodriguez@ehu.eus (A.R.-G.); 2 Dipartimento di Scienza e Tecnologia del Farmaco, Università degli Studi di Torino, via Pietro Giuria 9, 10125 Torino, Italy; elisabetta.muntoni@unito.it (E.M.); luigi.battaglia@unito.it (L.B.)

**Keywords:** gene therapy, solid lipid nanoparticles, polyvinyl alcohol (PVA), corneal inflammation, interleukin-10, transfection, IL-10 knock out mice, topical administration

## Abstract

One of the main challenges in gene therapy is the issue of delivery, and it is especially relevant for the success of gene therapy in the cornea. In the present work, eye drops containing biocompatible non-viral vectors based on solid lipid nanoparticles (SLNs) as gene delivery systems to induce the expression of interleukin 10 (IL-10) were designed to address the treatment of corneal inflammation. Two kinds of SLNs combined with different ligands (protamine, dextran, or hyaluronic acid (HA)) and formulated with polyvinyl alcohol (PVA) were prepared. SLN-based vectors were characterized in terms of size, adhesiveness, viscosity, and pH, before topical administration to wild type and IL-10 knock out (KO) mice. The formulations showed a homogenous particle size below 400 nm and a positive surface charge to favor bioadhesion; the incorporation of PVA improved the corneal penetration. After three days of treatment by topical instillation, SLN-based vectors mainly transfected corneal epithelial cells, HA-formulations being the most effective ones. IL-10 was capable of reaching even the endothelial layer. Corneal sections showed no histological change and formulations seemed to be well tolerated after repeated topical administration. These promising results highlight the possible contribution of non-viral gene augmentation therapy to the future clinical approach of corneal gene therapy.

## 1. Introduction

The cornea, a transparent anatomical structure in the anterior segment of the eye, plays a significant role in sight by refracting light to focus a visual image. This tissue can be injured by several factors (infections, dry eye, disorders of the eyelids, physical and chemical damage, and a wide variety of underlying diseases) causing corneal inflammation or keratitis [1]. Common symptoms and signs of keratitis include eye pain, blurred vision, photophobia, tearing, and eye redness, reaching visual impairment and blindness when chronic inflammation results in tissue destruction [2]. Current therapeutic management of keratitis shows limited efficacy, adverse effects, and short duration effect [3]. The advanced therapies, including gene therapy, are new rising approaches under evaluation.

Gene therapy medicinal products generally consist of a vector or delivery formulation/system containing a genetic construct engineered to express a specific transgene (‘therapeutic sequence’) for the regulation, repair, replacement, addition, or deletion of a genetic sequence [4]. These products are expected to have a significant impact on the biopharmaceutical market shortly [5]. A significant number of studies have evaluated the potential of gene therapy to deliver specific anti-inflammatory factors to treat keratitis. Interleukin-10 (IL-10) is a potent immunomodulatory cytokine that interacts with antigen presenting cells inhibiting the production of proinflammatory cytokines such as IL-1, IL-6, IL-8, and tumor necrosis factor (TNF)-alpha [1,6,7,8,9,10]. IL-10 gene delivery has been proposed to induce a sustained synthesis of the protein de novo in corneal cells, providing a long-term anti-inflammatory effect [11].

For the management of diseases in the anterior segment of the eye, topical administration is by far the most common route, although the low bioavailability of active molecules is an important limitation [12]. Formulations need to face lacrimal turnover, nasolacrimal drainage, blinking reflex, corneal barrier, and absorption of drugs by the conjunctiva [13,14]. An ideal delivery system for ocular topical administration should be easily administered, well tolerated with minimal side effects and with high retention time on the ocular surface to improve the penetration on the corneal tissue [13,15]. The design of delivery systems specifically adapted to the kind of the genetic material, the route, and way of administration and the target cell, is a key challenge for the clinical translation of non-viral gene therapy. Solid lipid nanoparticles (SLNs) are non-viral vectors composed of well-tolerated physiological lipids, which have previously demonstrated the capacity to transfect the epithelium, the stroma, and the endothelium of rabbit explanted corneas [11]. Due to their nanometer-range size, lipophilic properties, and usually cationic surface, SLNs can improve the ocular bioavailability of nucleic acids after topical administration, by means of enhancing the corneal penetration and the cellular uptake, extending ocular retention time and providing a controlled release profile [16,17,18,19]. Additionally, the physicochemical stability of SLNs makes possible their inclusion in solutions of viscosity modifiers widely used in ophthalmic eye drops, such as polyvinyl alcohol (PVA), in order to increase the residence time of the formulation in contact with the cornea and reduce drainage from lachrymal fluid [20,21]. 

The aim of the present work was the development, in vitro and in vivo evaluation in mice of four different gene therapy topical medicinal products to treat corneal inflammation. The formulations, based on two different types of SLNs, contained the plasmid encoding IL-10 to provide the de novo expression of this protein into the cornea. After optimization, the formulations were administered as eye drops to wild type mice and to IL-10 Knock Out (KO) mice, to evaluate the in vivo biodistribution and transfection capacity, and the ability to produce IL-10 in corneal tissues.

## 2. Materials and Methods 

### 2.1. Materials

DOTAP (1.2-Dioleoyl-3-trimethylammonium-propane chloride salt) was purchased from Avanti Polar-lipids Inc. (Alabaster, AL, USA), Tween 80 and dichloromethane from Panreac (Madrid, Spain), and sodium behenate from Nu-Chek Prep (Elysian, MN, USA). Precirol^®^ ATO5 was kindly provided by Gattefossé (Madrid, Spain), Natrosol™ 250 M pharma *M_w_* 720,000, 4500–6500 cps, and Oramix CG110 from Safic-Alcan (Barcelona, Spain).

Protamine sulfate salt Grade X (P), dextran (Mn of 3.26 KDa) (DX), DEAE-dextran, partially hydrolyzed PVA 9000–10,000 Da *M_w_* (PVA9000), PVA average *M_w_* 85,000–124,000, 87–89% hydrolyzed, Cell Counting Kit-8 (CCK-8) and IR-780 iodide were obtained from Sigma-Aldrich (Madrid, Spain). Hyaluronic acid (*M_w_* of 100 KDa) (HA) was acquired from Lifecore Biomedical and sodium hyaluronate cosmetic grade from Disproquima DSM (Barcelona, Spain). Plasmid pcDNA3-EGFP (6.1 kb) encoding the green fluorescent protein (GFP) was generously provided by the laboratory of Professor B.H.F. Weber (University of Regensburg, Regensburg, Germany). Plasmid pUNO1-hIL10 (3.7 kb), that encodes human IL-10, was acquired from InvivoGen (San Diego, CA, USA). 

Human Corneal Epithelial (HCE-2) cells were purchased from American Type Culture Collection (ATCC, Manassas, VA, USA) and reagents employed in HCE-2 cells culture, Dulbecco’s Modified Eagle´s Medium/Nutrient Mixture F-12 with GlutaMAX™ (DMEM/F-12 with GlutaMAX™), fetal bovine serum (FBS), attachment factor, trypsin-EDTA and penicillin-streptomicin, were obtained from Life Technologies (ThermoFisher Scientific, Madrid, Spain). EGF was acquired from Myltenyi Biotec (Madrid, Spain). ELISA for IL-10 and the DuoSet Ancillary reagent kit were obtained from R&D Systems (Minneapolis, MN, USA).

Triton X-100 and DNA from salmon sperm were purchased in Sigma-Aldrich (Madrid, Spain), DAPI-Fluoromount-G by Southern Biotech (Birmingham, AL, USA), paraformaldehyde (PFA) from Panreac, PBS and ProLong™ Diamond Antifade Mountant with DAPI were acquired from Gibco (ThermoFisher Scientific, Madrid, Spain).

GFP polyclonal antibody and goat anti-Rabbit IgG (H+L) Cross-Adsorbed Secondary Antibody Alexa Fluor 488 were purchased from Life Technologies (ThermoFisher Scientific, Madrid, Spain), rabbit Anti-IL-10 antibody and rat monoclonal CD44 antibody from Abcam (Cambridge, UK). Tissue-Tek^®^ O.C.T™ compound was obtained from Sakura Finetek Europe (Alphen aan den Rijn, The Netherlands). Other chemicals, if not specified, were reagent grade from Sigma Aldrich (Madrid, Spain) and Panreac (Barcelona, Spain).

### 2.2. Preparation of SLNs and Vectors

Two kinds of SLNs were prepared by different methods: solvent evaporation/emulsification (SLN_EE_) and by coacervation (SLN_C_).

SLN_EE_ consisted of a solid lipid core of Precirol^®^ ATO5, a cationic lipidic surface based on DOTAP together with the surfactant Tween 80, as previously published [22]. Briefly, DOTAP and Tween 80 were dissolved in water, then, this aqueous solution was mixed with Precirol^®^ ATO5 dissolved in dichloromethane, and the mixture was sonicated. Later, dichloromethane was evaporated. 

SLN_C_ were constituted by a lipid matrix of behenic acid, coated by PVA9000, as suspending agent, and DEAE-dextran as cationizing agent. For their preparation, behenic acid and PVA9000 were dissolved in water at 80 °C under stirring, and when the solution became translucent, NaOH was added, turning then transparent. DEAE-dextran was incorporated dropwise, and the mixture became turbid. Then, HCl was quickly added turning the suspension white, and, finally, it was cooled in a water bath under stirring. 

When necessary, IR780 iodide was incorporated in the preparation of both SLNs to label them. In the case of SLN_EE_, IR780 iodide was added together with Precirol^®^ ATO5, whereas in the case of SLN_C_ it was mixed at the end of the formation of the nanosuspension.

The vectors were formed at different weight to weight ratios (Table 1) as previously documented [23,24]. Briefly, the plasmid DNA (pcDNA3-EGFP or pUNO1-hIL10) was mixed with an aqueous solution of protamine (P) for 5 min; then, an aqueous solution of polysaccharide, dextran (DX) or hyaluronic acid (HA) was added and mixed for 15 min; finally, the suspension of SLNs was incorporated to the complexes previously obtained. 

In order to formulate the vectors with PVA, once the vectors were prepared, they were mixed with an aqueous solution of PVA (85,000–124,000 *M_w_* ) to a final concentration of 1% PVA. 

### 2.3. Size and Zeta Potential of SLNs and Vectors

SLNs and vectors were examined by dynamic light scattering, to determine size and polydispersity index, and by laser doppler velocimetry, to measure zeta potential. Samples were appropriately diluted in Milli-Q^TM^ water (EDM Millipore, Billerica, MA, USA) and analyzed using a Zetasizer Nano series-Nano ZS (Malvern Instruments, Worcestershire, UK, USA). Each measurement was carried out in triplicate.

### 2.4. Adhesion Test

An in vitro model developed by Gallarate et al. [25] was followed to simulate the flow rate of the formulations on the corneal surface, employing a gel with the same surface tension of tear fluid (28 dyne/cm). The gel was composed by 5% hydroxyethyl cellulose (Natrosol™ 250 M), 1% decylpolyglucoside (Oramix CG110), 3% HA cosmetic grade and water q.s. 100%. The gel was spread on a glass support until the appropriate hardness was achieved, then it was sloped 18°. A total of 100 µL of each sample was added on the top and the time needed for each drop to flow over a distance of 15 cm was measured. The assay was repeated three times with each formulation and the flow rate values were expressed as distance covered/time (cm/s).

### 2.5. Rheology Studies

Rheological behavior of the formulations was assessed employing an Advanced Rheometer AR1000 (TA Instruments, New Castle, DE, USA). The cone angle was 2° and the plate diameter was 40 mm. Measurements were carried out at room temperature. Rheology Advantage^TM^ software (TA Instruments, New Castle, DE, USA) was used to collect results. The shear stress and viscosity data were collected at shear rates from 5 to 1000 s^−1^ with 10 points per decade and fitted to the power law model:

τ = k (γ)n,(1)

where τ is the shear stress (Pa), γ∙is the shear rate (s^−1^), k is the consistency coefficient (Pa·s^n^), and n is the flow behavior index. In order to study the overall flow characteristics, the logarithm of shear stress versus logarithm of shear rate was plotted:

log τ = log k + n log γ.(2)

The value of n indicates the rheological behavior of the fluid: n value lower than 1 indicates a shear-thinning behavior, and it is classified as pseudoplastic; if n is 1, the rheological behavior is Newtonian; and when n is greater than 1, it is dilatant. The value of k helps to figure out the viscosity when fluids have similar flow behavior index [26,27,28].

Viscosity values (mPa s) were plotted to evaluate the behavior at the different shear rates, and then compared at 10 and 500 s^−1^.

### 2.6. pH Measurement

pH of the vectors and plasmid solutions (with or without PVA 1%) were determined in triplicate employing a Crison Basic 20 pH meter (Crison Instruments, Barcelona, Spain), which was calibrated daily.

### 2.7. In Vitro Studies

For in vitro studies HCE-2 cells were cultured in DMEM/F-12 with GlutaMAX™ supplemented with 15% of fetal bovine serum, 4 mg/mL of insulin, 10 ng/mL of EGF, and 1% of penicillin-streptomycin. Cells were incubated at 37 °C with 5% CO_2_ and sub-cultured every 7 days (at 80% of confluence) in flasks previously treated with 4 mL of Attachment Factor. 

#### 2.7.1. Transfection Efficacy of the Vectors Containing the Plasmid pUNO1-hIL10 

Cells were seeded on 24-well plates previously incubated with Attachment Factor at a density of 150,000 cells/well, and allowed to adhere and create a monolayer for 72 h. Then, cells were treated with 75 µL of each vector (2.5 μg of pUNO1-hIL10 plasmid) for 4 h. After the incubation period, the medium with the vectors was removed and cells were allowed to grow 72 h more. 

In order to quantify IL-10 levels, an Enzyme-linked Immunosorbent Assay (ELISA) was used. The medium of the wells was removed and centrifuged, then 100 µL of the supernatant was added to a 96-well plate and secreted IL-10 was measured following the manufacturer’s instructions of the ELISA kit.

#### 2.7.2. In Vitro Cell Viability 

Cells were seeded in a 96-well plate at 5 × 10^3^ cells per well, after incubation of the plate with Attachment Factor, and allowed to grow for 24 h. Then, HCE-2 cells were exposed to 10 µL of the formulations and to a positive control (10% Triton X-100 in PBS solution). Four hours later, vectors were removed, and fresh medium was added to the wells. Cell cytotoxicity was evaluated 72 h later, after 4 h of incubation with 10 µL of CCK-8 (water soluble tetrazolium salt, WST-8), employing a microplate reader with a wavelength of 450 nm (Glomax^®^-Multi Detection System (Promega Corporation, Madison, WI, USA), following manufacturer’s instructions. The percentage of viable cells was expressed as percentage respect to untreated cells. 

### 2.8. In Vivo Studies

Six-week-old male C57BL/6 mice and IL-10 KO mice (JAX stock #002251) [29] acquired from The Jackson Laboratory were employed for the in vivo studies with a weight between 20 and 25 g. These experiments were approved by the Animal Experimentation Ethics Committee of the University of the Basque Country UPV/EHU (license M20/2018/142) following the Spanish and European Union (EU) laws and all the procedures were followed in accordance. Before the experiments started, mice were allowed to acclimatize. Animals possessed food and water ad libitum and they were maintained under controlled temperature, humidity, and day-night cycles. 

In order to avoid distress during experimental manipulation, mice were anesthetized with 1–2% isoflurane (IsoFlo, Abbott, Madrid, Spain) in air, at a flow rate of 0.5–1 L/min. 

Animals were humanely euthanatized by cervical dislocation. Eyeballs were removed, washed in physiological saline solution, and fixed with 4% PFA during 30 min. Later on, they were washed with PBS for 5 min and incubated at 4 °C with 30% sucrose in PBS until the eye was sunk. Next day, half of the volume was replaced with Tissue-Tek^®^ O.C.T™ compound and rocked at room temperature for 2 h. Finally, eyeballs were embedded in a mold with 100% Tissue-Tek^®^ O.C.T™ compound, frozen at −80 °C, and histological sections of 14 µm were made on a cryostat (Cryocut 3000, Leica, Bensheim, Germany) for further studies.

#### 2.8.1. Detection of CD44 Receptor in Cornea from Wild Type and IL-10 KO Mice

The presence of the CD44 (receptor for HA) in wild type and IL-10 KO mice corneas was studied by immunofluorescence. Cryosections were washed with PB buffer, blocked and permeabilized with a blocking solution (20% PB buffer, 0.3% Triton X-100, 10% goat serum, and water q.s. 100%) for 30 min. Later, cryosections were incubated for 24 h at 4 °C with the rat monoclonal CD44 antibody. Then, after washing the samples with PB, they were incubated with Alexa Fluor 488-conjugated goat anti-rat IgG for 30 min protected from the light. Finally, after a washing period, samples were dried and mounted with DAPI-Fluoromount-G. Tissue sections were examined under a Zeiss LSM800 confocal microscope. Sequential acquisition was used to avoid overlapping of fluorescent emission spectra. From each cornea, six sections representing the whole tissue were analyzed. Technical and human support for confocal microscopy was provided by the General Service (SGIker) of Analytical Microscopy and High Resolution in Biomedicine at the University of the Basque Country UPV/EHU.

#### 2.8.2. Corneal Localization of the Vectors

In order to follow the presence of the vectors in the cornea, the following formulations were topically administered on the ocular surface of wild type mice; DX-SLN_EE_, HA-SLN_EE_, and HA-SLN_C_, all of them with and without PVA. For this purpose, we prepared the vectors with IR780 iodide dyed SLNs and the plasmid pcDNA3-EGFP.

Three instillations of 2.5 µL of the formulations were administered employing a micropipette to one eye in each animal (the other eye was kept as control) at 3 min intervals, twice separated by 12 h. Mice were sacrificed 2 h after last dose and eyeballs were removed and treated as explained above. Three animals per formulation were evaluated.

Sections were mounted with ProLong™ Diamond Antifade Mountant with DAPI, and were examined under a Zeiss LSM800 confocal microscope (ZEISS microscopy, Oberkochen, Germany). Sequential acquisition was used to avoid overlapping of fluorescent emission spectra. From each cornea, six sections representing the whole tissue were analyzed. 

#### 2.8.3. In Vivo Transfection Studies

##### Topical Administration 

To evaluate the capacity of the vectors to transfect the cornea, we administered topically on the ocular surface of wild type mice the following formulations with the plasmid pcDNA3-EGFP: DX-SLN_EE_, HA-SLN_EE_, and HA-SLN_C_, with and without PVA. Additionally, we also administered naked plasmid with and without PVA. A total of 4.5 µg of DNA per day were administered, separated in two doses over 3 days. Each dose consisted of three instillations of 2.5 µL at 3 min intervals. 

We also evaluated the vectors containing the plasmid pUNO1-hIL10 in both wild type and in IL-10 KO mice. The animals were treated following the same dosing protocol described above with the following formulations: DX-SLN_EE_ with PVA, HA-SLN_EE_ with PVA, and HA-SLN_C_ with PVA. Naked plasmid with PVA was also studied.

Three animals per formulation were evaluated, and in each animal, one eye was treated and the other one was kept as control.

##### Evaluation of Gene Expression

Forty-eight hours after the last dose, mice were sacrificed and eyeballs were removed, fixed, and sectioned as explained above. Immunofluorescence staining was performed to evaluate GFP or IL-10 expression, qualitatively. Slides containing the sections were washed with PB buffer, blocked, and permeabilized employing a solution of 20% PB, 0.3% Triton X-100, 10% goat serum, and water q.s. 100% for 30 min. Later, primary antibody anti-GFP or anti-IL-10 was incubated 24 h at 4 °C. After that, samples were washed, and secondary antibody goat anti-rabbit IgG Alexa Fluor 488 was added for a 30 min incubation protected from light. After a washing period, samples were dried and mounted with DAPI-Fluoromount-G. Tissue sections were examined under a Zeiss LSM800 confocal microscope (ZEISS microscopy, Oberkochen, Germany). Sequential acquisition was used to avoid overlapping of fluorescent emission spectra. From each cornea, six sections representing the whole tissue were analyzed.

##### Structural Analysis of the Cornea

The structure of the corneal sections of wild type and IL-10 KO mice was analyzed by the Masson’s trichrome staining technique, before and after treatment with the formulations. Samples were observed in a Leica DM IL LED Fluo inverted microscope (Leica Microsystems CMS GmbH, Wetzlar, Germany).

### 2.9. Statistical Analysis

Statistical analysis was performed with IBM SPSS Statistics 23 (IBM) software, employing Saphiro–Wilk test for evaluation of normal distribution of samples and Levene test for homogeneity and variance. ANOVA or Student’s *t*-test were used for comparisons and *p* < 0.05 was considered statistically significant. 

## 3. Results

### 3.1. Size and Zeta Potential of SLNs and Vectors

Table 2 shows the mean diameter, PDI, and zeta potential of the two kind of SLNs and the vectors bearing the plasmid pcDNA3-EGFP or the plasmid pUNO1-hIL10. Particle size of SLN_C_ resulted as significantly higher than that of SLN_EE_ (453.9 ± 13.6 nm vs. 202.2 ± 28.2 nm), and both presented positive charge and PDI lower than 0.3, which indicates homogeneity in the particle size.

The size of the vectors ranged from 159.4 to 447.9 nm, and zeta potential from +14.7 to +39.3 mV. DX-SLN_EE_ vectors, regardless of the plasmid used, presented smaller size and higher zeta potential than the other vectors. The highest size corresponded to DNA-SLN_C_ vectors. Moreover, when the plasmid pUNO1-hIL10 was used, the zeta potential significantly decreased in the vectors DNA-SLN_C_ and HA-SLN_C_ (+14.7 ± 0.7 and +15.8 ± 2.4, respectively), with respect to the same vectors containing the plasmid pcDNA3-EGFP.

There was no statistically significant change in particle size, PDI, and zeta potential of SLNs and vectors labelled with IR 780 iodide.

### 3.2. Adhesion Test: Flow Rates

**Figure 1:**
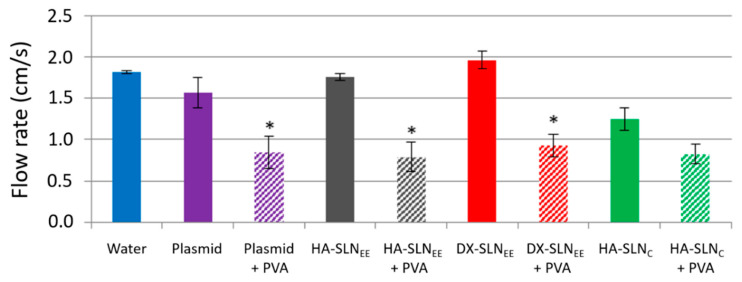
Flow rates of water, plasmid solution, SLNs, and vectors with and without PVA (expressed as cm/s). * *p* < 0.05 with respect to the same formulation without PVA. PVA: polyvinyl alcohol; HA: hyaluronic acid; DX: dextran; SLN: solid lipid nanoparticle.

An adhesion test was performed in order to study the behavior of the formulations in terms of flow rates; the higher the flow rate the lower the adhesiveness. As it can be observed in Figure 1*,* the solution of plasmid pcDNA3-EGFP, and HA-SLN_EE_ and DX-SLN_EE_ vectors showed a flow rate similar to that of water. The addition of PVA decreased the flow rate, which means that it increased the adhesiveness of the plasmid solution and all the formulations, except for HA-SLN_C_ vector, which did not show significant differences in the flow rate with or without PVA. The flow rate values obtained with the plasmid pUNO1-hIL10 did not show significant differences with respect to these presented in Figure 1. 

### 3.3. Rheology Studies

Table 3 summarizes the results obtained in the rheological studies. In all cases, when plotting the logarithm of shear stress versus logarithm of shear rate, high coefficient of determination values (R^2^) were obtained. 

**Figure 2:**
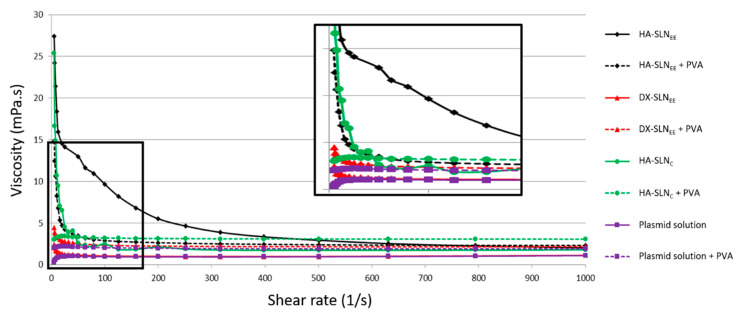
Viscosity curve at shear rates from 5 to 1000 s^−1^ of water, the solution of plasmid pcDNA3-EGFP and vectors, with and without PVA. PVA: polyvinyl alcohol; HA: hyaluronic acid; DX: dextran; SLN: solid lipid nanoparticle.

pcDNA3-EGFP plasmid solution, with and without PVA, and HA-SLN_C_ with PVA showed a flow behavior similar to water, with a n index near 1, indicating Newtonian behavior, where viscosity is constant independently of the shear rate applied. By contrast, all the SLN_EE_-based vectors, with and without PVA, and the vector HA-SLN_C_ (without PVA) showed lower values of n, which indicates a pseudoplastic (shear-thinning) behavior. In Figure 2 the decrease in the apparent viscosity of these vectors as shear rate increased can be observed.

As it is observed in Table 3 and Figure 2, at low shear rate values, PVA increased the apparent viscosity of plasmid solution and DX-SLN_EE_ vectors, whereas in the case of the vectors containing HA the viscosity was much higher without PVA. At high shear rates, PVA increased or maintained the viscosity of HA-containing formulations. The results corresponding to the plasmid pUNO1-hIL10 and the vectors prepared with it are included in Supplementary Materials.

### 3.4. pH Values

Table 4 features the pH values of the plasmid solutions and the vector suspension, with and without PVA. The results correspond to the plasmid pcDNA3-GFP; the pH values obtained with the plasmid pUNO1-hIL10 did not show significant differences respect to these presented in Table 4. The pH of the plasmid and the vectors prepared with SLN_EE_ ranged from 7.5 to 7.0; however, the vectors prepared with SLN_C_ presented a lower pH, around 4.0. In all cases, the PVA hardly modified the pH. 

### 3.5. In Vitro Studies

#### 3.5.1. Transfection Efficacy of the Vectors Containing the Plasmid pUNO1-hIL10 

In order to study the capacity of HA-SLN_C_ bearing the pUNO1-hIL10 plasmid to induce the expression of IL-10, HCE-2 cells were treated with the vector. Seventy-two hours later the concentration of IL-10 in the culture medium was 9.1 ± 0.8 ng/mL. This level was similar to that of DX-SLN_EE_ and HA-SLN_EE_, which we assessed in a previous study [11]. In the culture medium of untreated cells and the cells treated with the free plasmid and with the plasmid condensed with protamine and the HA or DX (without SLNs), IL-10 was not detectable. 

#### 3.5.2. In Vitro Cell Viability

**Figure 3:**
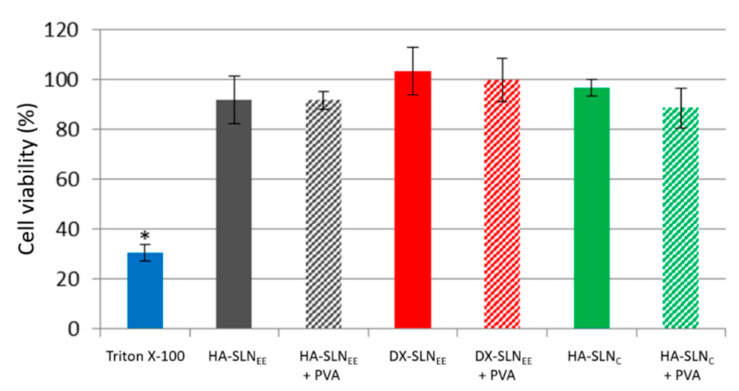
Cell viability after treatment of HCE-2 cells with Triton X-100 as positive control, and with the following formulations: HA-SLN_C_, HA-SLN_C_ with PVA, DX-SLN_EE_, DX-SLN_EE_ with PVA, HA-SLN_EE_, and HA-SLN_EE_ with PVA. The percentage of viable cells was expressed as percentage respect to untreated cells. * *p* < 0.01 respect to the formulations. HA: hyaluronic acid; PVA: polyvinyl alcohol; DX: dextran; SLN: solid lipid nanoparticle; HCE-2 cells: human corneal epithelial cells. *n* = 3; data are expressed as mean ± standard deviation.

The viability of the HCE-2 cells after the treatment with the formulations was assessed by using the CCK-8 assay. As shown in Figure 3, in all cases cell viability of the HCE-2 cells was higher than 80%, except for the positive control Triton X-10, which reduced the cell viability to 30.51% ± 3.30%.

### 3.6. In Vivo Studies

#### 3.6.1. Detection of CD44

**Figure 4:**
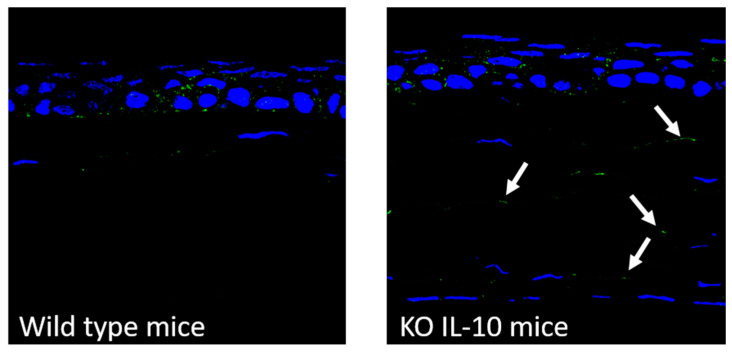
CD44 detection by immunofluorescence in corneal tissue from wild type and IL-10 KO mice (20×). Blue: nuclei stained with DAPI. Green: CD44. KO: knock out; IL-10: interleukin-10.

Figure 4 shows the expression of the CD44 receptor (green color) in the corneal tissue of wild type and IL-10 KO mice. As can be seen, in the cornea of wild type animals, CD44 was detected only in the epithelial layers, whereas in the corneas of IL-10 KO mice, CD44 was detected in epithelium and also in the stroma (arrows). 

#### 3.6.2. Corneal Localization of the Vectors

**Figure 5:**
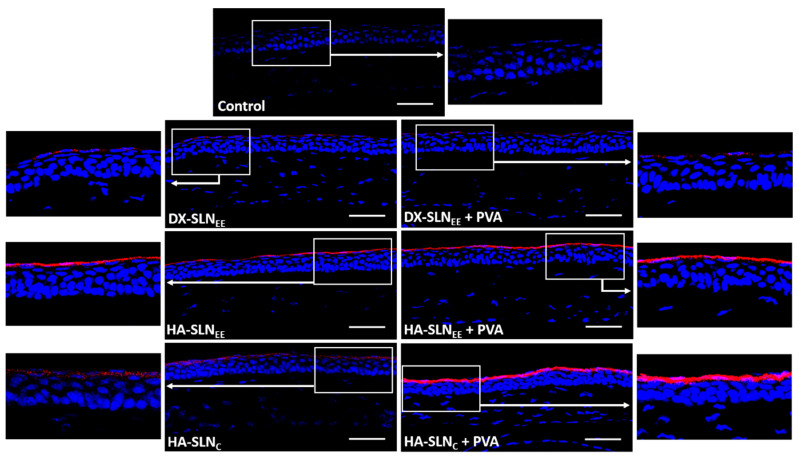
Localization of the formulations on the cornea of wild type mice (20×), 2 h after the instillation of the last dose. Blue: nuclei stained with DAPI. Red: vectors with IR780 iodide labelled SLNs. Scale bar: 50 µm. DX: dextran; SLN: solid lipid nanoparticle; PVA: polyvinyl alcohol; HA: hyaluronic acid.

In order to study the corneal localization of the formulations (with and without PVA), they were prepared with IR780 iodide labelled SLNs (red) and administered topically on the ocular surface of wild type mice. Figure 5 features images of the corneas 2 h after the instillation of the last dose. All the vectors were localized on the corneal epithelium.

When HA-SLN_EE_, prepared with 2.5 times less particles than DX formulations for the same DNA dose, was administered, higher fluorescence intensity was observed, regardless of the presence of PVA. This higher fluorescence intensity indicates a higher amount of HA-SLN_EE_ vectors in the corneal epithelium than in the case of DX-SLN_EE_. On the contrary, HA-SLN_C_ vectors, although prepared with a higher SLN_C_:DNA ratio (10:1) than in SLN_EE_-based vectors, presented a lower fluorescence intensity, which is indicative of a lower amount of vectors in the cornea. The incorporation of PVA increased the fluorescence intensity on the cornea of the SLN_EE_- and SLN_C_-based vectors formulated with HA as polysaccharide. However, the combination of DX-SLN_EE_ with PVA did not modify the corneal localization and fluorescence intensity observed.

#### 3.6.3. In Vivo Transfection with the Vectors Containing the Plasmid pcDNA3-EGFP 

**Figure 6:**
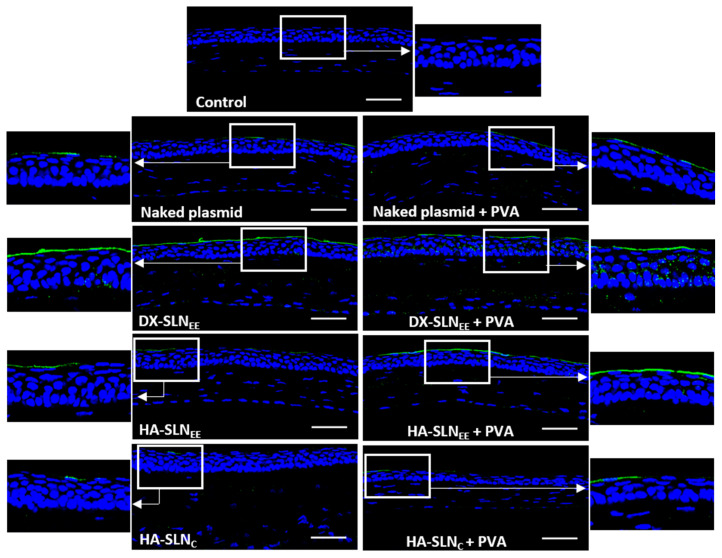
Corneal transfection in vivo in wild type mice treated with naked plasmid and vectors bearing the plasmid pcDNA3-EGFP with and without PVA (20×). Blue: nuclei stained with DAPI. Green: green fluorescent protein. Scale bar: 50 µm. DX: dextran; SLN: solid lipid nanoparticle; PVA: polyvinyl alcohol; HA: hyaluronic acid.

In order to assess the location of the transfected corneal cells, the vectors bearing the plasmid pcDNA3-EGFP were topically administered to wild type mice. This plasmid encodes the reporter GFP, which is an intracellular protein. Transfection results are summarized in Figure 6.

GFP was detected in the 100% of the sections analyzed. All the formulations were able to transfect the epithelial cells, although in the corneas of mice treated with naked DNA and with HA-SLN_C_ (with or without PVA), GFP was localized in a discontinuous way. 

GFP produced by the DX-SLN_EE_ was detected mainly in the surface of the epithelium, but the protein was also observed in inner epithelial layers, whereas HA-SLN_EE_ and HA-SLN_C_ transfected only the outermost layer of the corneal epithelium. 

No difference was observed in transfection between the naked plasmid with and without PVA. However, PVA notably affected the transfection capacity of the vectors. On the one hand, DX-SLN_EE_ with PVA induced higher expression in deeper layers of the epithelium; on the other hand, HA-SLN_EE_ and HA-SLN_C_ combined with PVA resulted in higher fluorescence intensity, which is associated to a higher protein expression. 

#### 3.6.4. In Vivo Transfection with the Vectors Containing the pUNO1-hIL10 Plasmid

**Figure 7:**
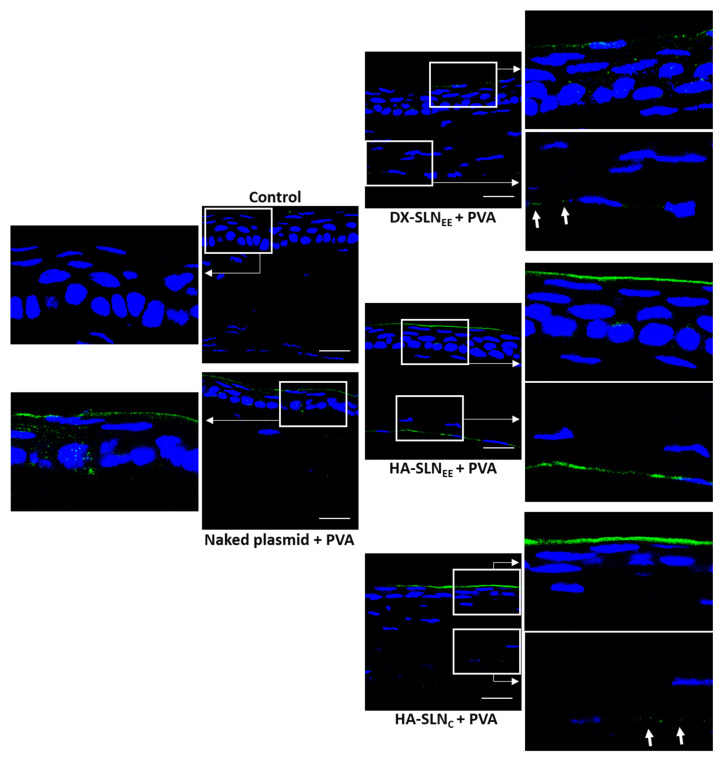
Corneal transfection in vivo in wild type mice treated with naked plasmid and vectors bearing the plasmid pUNO1-hIL10 with PVA (60×). Blue: nuclei stained with DAPI. Green: IL-10. Scale bar: 20 µm. For each sample, a global image of the corresponding corneal section and a magnification of the epithelium and endothelium areas have been included. Arrows in magnifications indicate detection of IL-10. DX: dextran; SLN: solid lipid nanoparticle; PVA: polyvinyl alcohol; HA: hyaluronic acid.

**Figure 8:**
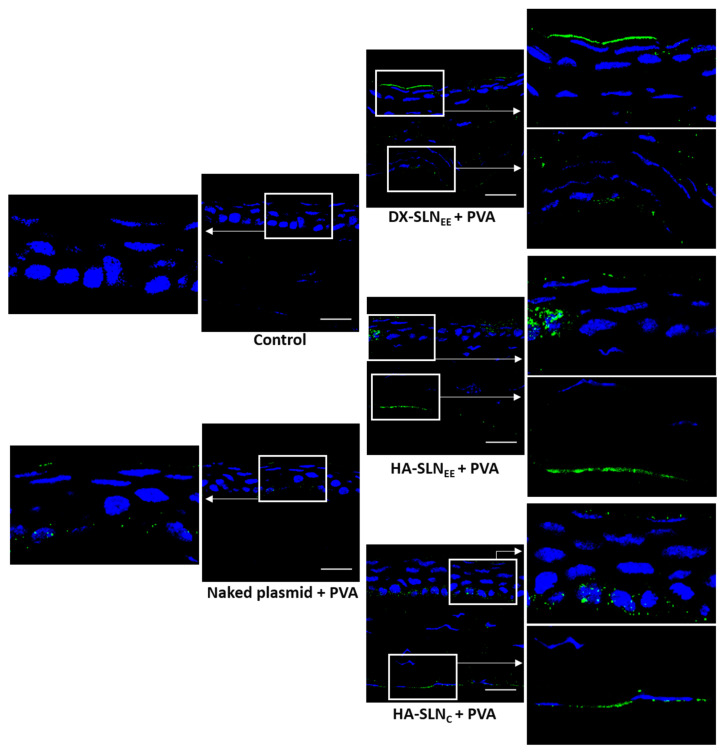
Corneal transfection in vivo in IL-10 KO mice treated with naked plasmid and vectors bearing the plasmid pUNO1-hIL10 with PVA (60×). Blue: nuclei stained with DAPI. Green: IL-10. Scale bar: 20 µm. For each sample, a global image of the corresponding corneal section and a magnification of the epithelium and endothelium areas have been included. Arrows in magnifications indicate detection of IL-10. DX: dextran; SLN: solid lipid nanoparticle; PVA: polyvinyl alcohol; HA: hyaluronic acid.

The ability of the vectors combined with PVA to express IL-10 was assessed qualitatively in wild type mice (Figure 7) and IL-10 KO mice (Figure 8) after topical administration. Both wild type and IL-10 KO treated mice showed IL-10 transfection in the 100% of the sections analyzed, representative of the whole cornea. 

In the corneas of wild type mice (Figure 7) treated with naked plasmid and DX-SLN_EE_, IL-10 was detected in a discontinuous way (as dots), whereas in the corneas treated with HA-SLN_EE_ and HA-SLN_C_, the presence of the protein was continuous along the cornea. A higher intensity of fluorescence, indicative of higher protein synthesis, was observed in the corneas treated with HA-SLN_EE_ and HA-SLN_C_ formulations. In addition, the secreted IL-10 was even detected in the endothelial layer of the corneas treated with SLN_EE_-based vectors, especially when HA-SLN_EE_ vector was administered. After administration of DX-SLN_EE_ and HA-SLN_C_ vectors, the fluorescence corresponding to the IL-10 in the endothelial layer appeared in a dotted form (arrows in magnifications in Figure 7).

In IL-10 KO mice (Figure 8) the location profile of the secreted IL-10 was quite similar with the three vectors. IL-10 was detected in both the epithelium and the endothelium, and as in wild type mice, the intensity of fluorescence was higher in the corneas treated with the formulations containing HA as polysaccharide. 

#### 3.6.5. Structural Analysis of the Cornea

**Figure 9:**
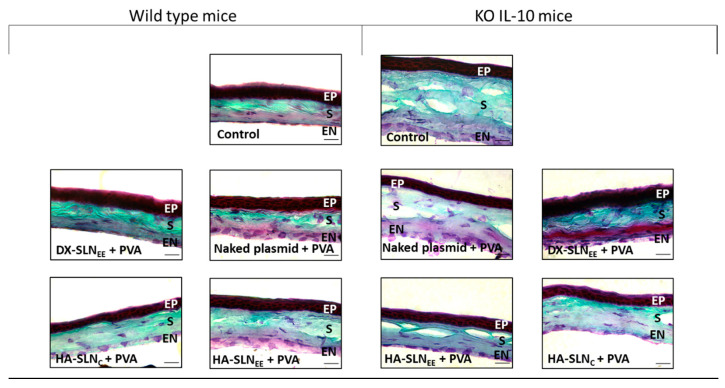
Microscopic images of wild type and IL-10 KO mice corneas stained by Masson’s trichrome technique. (60×) Scale bar: 20 µm. Green: collagen. Red: cell cytoplasm. Dark brown: cell nuclei. EP: epithelium, S: stroma, EN: endothelium; DX: dextran; SLN: solid lipid nanoparticle; PVA: polyvinyl alcohol; HA: hyaluronic acid; KO: knock out; IL-10: interleukin-10.

Figure 9 depicts histological sections of the corneas from wild type and IL-10 KO mice treated with the naked pUNO1-hIL10 plasmid and with the vectors containing the plasmid and combined with PVA, as well as untreated corneas (control).

The histological structure of the corneas from IL-10 KO mice showed differences with respect to those from wild type mice. The stroma of the untreated eyes of IL-10 KO mice showed gaps that were not present in the wild type corneas. 

The corneas of wild type mice treated for 3 days with the formulations did not show changes in the histological structure respect to the non-treated corneas. Therefore, formulations seem to be well tolerated after repeated topical administration.

## 4. Discussion

The clinical application of nucleic acid medicinal products is closely dependent on the development of effective and safe delivery systems, which must be specifically adapted to the characteristics of the genetic material and to the target tissue. In fact, the issue of delivery is considered as the main challenge in gene therapy and it is especially relevant for the success of gene therapy in the cornea. 

The most efficient methods for nucleic acid-based therapies are viral vectors. Retroviruses, lentiviruses, adenoviruses, and adeno-associated viruses have been used for transfecting the cornea. However, the induction of the immune response and inflammation limits the application of viral vectors to inflammatory diseases, including cornea inflammation, even considering its relative immune privilege [1]. Moreover, they have been frequently administered by invasive methods such as intrastromal, intralimbal, intracameral, or after removing the corneal epithelium [30,31,32]. Non-viral gene therapy has proven to be a feasible alternative for corneal gene therapy even after topical administration [33,34,35,36]. Non-viral vectors assessed for topical corneal gene therapy include, among others, magnetic nanoparticles [37], hyaluronan/chitosan nanoparticles [33], hybrid nanoparticles based on gelatin and chondroitin sulphate [38], polyethylenimine [39], gold nanoparticles [35], polyethylenimine-conjugated gold nanoparticles [40], and SLNs [34], but according to the results of those works there is still room for improvement. Likewise, corneal inflammation is usually associated to pathologies that occur in outbreaks and it does not require the long-term expression of anti-inflammatory mediators. In this sense, topical instillations of non-viral vectors could be self-administered by the patients themselves when symptoms appear. In the present work, we formulated eye drops containing biocompatible non-viral vectors based on SLNs capable of increasing ocular bioavailability of IL-10 in different corneal layers after topical administration, as an in vivo proof of concept of the utility of gene therapy to address the treatment of corneal inflammation.

The nanovectors were prepared with two different kinds of cationic SLNs: SLN_EE_, prepared by solvent evaporation/emulsification, and SLN_C_, prepared by coacervation; this second method avoids the use of solvents. The final vectors also contained ligands on their composition, a cationic peptide, protamine, and a polysaccharide, DX or HA, which confers to the vectors a high versatility. On the one hand, the positive charge of SLN facilitates the interaction with the polyanionic corneal surface, increasing the retention time and improving corneal permeation through endocytic uptake by corneal epithelial cells [17,41]. On the other hand, polysaccharides determine the interaction with targeted cells, and as a consequence, the internalization process and the intracellular behavior of the genetic material [34,42]. Finally, protamine, apart from favoring the transcription process and nuclear entry, also contributes binding and protecting the genetic material at intra and extracellular level, thanks to its cationic nature [43]. 

The particle size of SLN_C_ (Figure S1 and Table 2), was more than double than that of SLN_EE_ (453.9 ± 13.6 nm vs. 202.2 ± 28, respectively); accordingly, SLN_C_-based vectors presented higher particle sizes. Particle size of all the vectors ranged from 159.4 to 447.9 nm and the PDI were lower than 0.4, indicating homogeneity in the particle size. Nanoparticles smaller than 800 nm avoid ocular irritation or discomfort and favor uptake by corneal cells [44,45,46]. In addition, nanoparticles with an average diameter ranging from 50 to 400 nm have the ability to overcome physiological barriers, and they lead to higher bioadhesion and corneal penetration when they are topically administered [47,48,49,50]. In fact, patients tolerate smaller particles better than larger ones, because the former are more able to penetrate across the corneal barrier [51]. In the present work, DNA-SLN_C_ vector was the only one with size above 400 nm. Contrary to the others, this vector was prepared without protamine, a peptide known by their ability to condense DNA, which has been directly related with a reduction in size [43]. Hence, the DNA-SLN_C_ vector was discarded for the following studies, although all SLN_C_-based vectors were able to fully bind and protect the genetic material, and to release the DNA although not completely (Figure S2).

Electrostatic interactions between the components of the formulation determine the final structure and the physicochemical characteristics of the vectors. All SLN-based products had a positive surface charge (ranging from +14.7 to +39.3 mV) which promotes their interaction and retention in the cornea. The inner layer of the tear film is the mucous network. This layer is in contact with the corneal epithelium and presents sialic acid and sulfate residues responsible of the negative charge of mucin at physiological pH, which favors the uptake of cationic molecules [13,52]. 

Once the vectors were obtained, PVA, a viscosity-enhancing agent, was added to a final concentration of 1% with the aim of improving corneal retention and ocular bioavailability. PVA is a non-ionic synthetic linear and hydrophilic polymer, widely employed in ophthalmic preparations because of its biocompatibility, the lack of interaction with many active compounds, and its capacity to improve the ocular absorption [53]. It is even a component of contact lenses and artificial corneas [28,54,55]. In artificial tears, ocular lubricants, and drug-containing eye drops PVA is normally used at a concentration of 0.5–1.4% [52].

Corneal adhesion of the formulations was evaluated with a previously reported in vitro model [25]. While the flow rates of a free solution of the plasmid and the SLN_EE_-based vectors (DX-SLN_EE_ and HA-SLN_EE_) were similar to that of water, a statistically significant decrease was observed in the flow rate values after the addition of PVA to these formulations (Figure 1). Therefore, this polymer provides an enhancement in the adhesiveness properties that would raise their residence time on the corneal surface. PVA did not modify the flow rate values of HA-SLN_C_, which were similar to that obtained for SLN_EE_ formulations combined with PVA.

Rheological studies were conducted in order to obtain complementary information about viscosity of the formulations as well as their flow behavior. A high viscosity can result in discomfort due to blurred vision and foreign body sensation, leading to a faster elimination for reflex tears and blinks [56,57]. All SLN formulations without PVA, as well as those with PVA, showed a pseudoplastic behavior, except for HA-SLN_C_. For DX-SLN_EE_ the pseudoplastic behavior was less evident, and the viscosity effect of PVA could be better appreciated. Indeed, a slight increase in the viscosity values was observed in DX-SLN_EE_ vectors when they were combined with PVA. On the contrary, the viscosity of the formulations prepared with HA was much higher without PVA (Table 3), maybe related to the inherent viscous and mucoadhesive properties of the HA [13,46,56]. The HA formulations without PVA were markedly pseudoplastic, but the rheological behavior in the presence of PVA was different depending on the type of SLN. PVA solutions have a Netwonian behavior by themselves [58]. When PVA was added to the HA-SLN_C_ vector the rheological behavior became Newtonian, whereas the combination of PVA with the HA-SLN_EE_ vector resulted in pseudoplastic behavior, although n increased and the change on viscosity from low to high shear rates was less marked. 

Since tear fluid possesses pseudoplastic properties, topically administered solutions with the same rheological behavior will be more advantageous [14]. Indeed, at low shear rate, the high viscosity improves retention time, avoiding drainage [26,53,59]. During blinking, which involves high shear rate, instead, decrease of viscosity allows the formulations to spread over the corneal surface, offering less resistance to blinking, making them well accepted [14,53,60]. However, PVA confers a certain viscosity at high shear rate, improving the corneal retention also during the blinking phase.

Another requirement for ophthalmic preparations is an appropriate pH. The ideal pH is as close as possible to tears (7.0–7.5) to avoid discomfort [61,62]. However, pH values between 4 and 8 are well tolerated by the eye thanks to the buffering capacity of the tears, which prevents eye irritation [14,53,63]. SLN_EE_ -based formulations showed pH values from 7.3 to 7.5, while the pH of SLN_C_-based vectors was 4.0, due to the preparation method which is pH-dependent [64]. The formulation of the vectors with PVA hardly modified the pH values (Table 4). The administration of the formulations on the ocular surface of the mice showed no sign of irritation on the external ocular tissues. Moreover, Masson’s trichrome staining of the corneal sections did not show histological changes after 3 days of treatment in both, wild type and IL-10 KO mice, with respect to the non-treated corneas (Figure 9).

Apart from the technological properties of the formulations relevant for a certain administration route, gene medicinal products need to demonstrate efficacy in terms of transfection capacity. Once inside the cell, the genetic material has to overcome different barriers for a successful transfection, including escape from endocytic vesicles, diffusion through the cytoplasm, and transport into the nucleus for transcription process [34,42,65]. SLN-based vectors have previously demonstrated their capacity to enter and release the plasmid into corneal cells [11]. In the present work, we firstly evaluated in vitro the capacity of the HA-SLN_C_ vector to induce the production of the therapeutic protein IL-10 in HCE-2 cells. HA-SLN_C_ formulation was able to induce the production IL-10, achieving similar concentrations to that previously obtained with SLN_EE_ vectors [11].

In addition to the ability to overpass the limiting barriers at intracellular level, the vectors must be able to overcome all the accessibility issues, providing an adequate disposition of the genetic material in the target cells. The multi-component nanosystem developed here, when administered topically to mice as eye drops, remained on the corneal epithelium at least 2 h after the last dose (Figure 5). Differences in the distribution were detected depending on the composition of the vector, but also depending on the presence of PVA. The HA-SLN_EE_ vector seems to be the most effective to overcome corneal barriers, since, although it contains a lower amount of SLNs, higher fluorescence intensity was observed into the corneal epithelial cells. CD44 may be involved in the internalization process of the formulations containing HA [42,66]. CD44 is a receptor able to interact with the HA; it has been found under normal conditions in basal and apical layers of the cornea, and its expression is increased during injury [67]. In fact, Figure 4 shows CD44 expression in the epithelial layer of wild type mice. The incorporation of PVA to the vectors formulated with HA increased the fluorescence signal, indicative of a higher retention on the cornea. When both HA and PVA are included in the final formulation, the adhesiveness properties and the decrease in viscosity seem to facilitate the ability of the vectors to penetrate the cornea. The distribution of the vectors matches with the distribution of GFP (Figure 6), indicative of the cells that were transfected, since GFP is an intracellular protein. Both HA-SLN_EE_ and HA-SLN_C_ with PVA showed higher GFP expression in corneal epithelium. Biopharmaceutics plays a significant role in the design and evaluation of gene therapy medicinal products. In our study, gene delivery systems were able to induce the production of the protein in the stratified and renewable epithelial layer, which is advantageous due to the high number of cells that can be transfected for the production of high levels of the protein. By contrast, gene expression in other corneal layers such as endothelium, which contains a low number of cells and a complicated accessibility, would lead to lower levels of protein. However, considering the secreted nature of IL-10, its presence in different corneal cells, not only in endothelial ones, is expected; from the therapeutic point of view, it is beneficial, since the anti-inflammatory effect of this cytokine will not be restricted to the transfected cells. The incorporation of PVA did not influence the transfection capacity of the naked plasmid, but it increased the efficacy of all vectors; hence, PVA-formulations were selected for the following studies with the vectors bearing the plasmid pUNO1-hIL10. IL-10 is a potent anti-inflammatory cytokine, but its therapeutic use is limited due to biopharmaceutical issues, mainly low ocular bioavailability and short half-life. The beneficial effect of IL-10 in ocular diseases have been shown in promoting corneal transplant survival [68], and in herpetic keratitis models [69] including IL-10 deficient mice [70]. We evaluated the ability of the vectors containing PVA to induce the expression of the IL-10 de novo into the cornea in wild type and in IL-10 KO mice. After 3 days of topical treatment, transfection was detected in the 100% of the sections analyzed.

In wild type mice (Figure 7), HA-SLN_EE_ and HA-SLN_C_ provided the highest intensity of fluorescence indicative of a raised IL-10 synthesis; these results are consistent with the localization and transfection pattern with vectors bearing the plasmid pcDNA3-EGFP. With all vectors, IL-10 was detected not only in the epithelium but also in the endothelial layer, the intensity of fluorescence being significant higher in the endothelium of corneas transfected with the HA-SLN_EE_. Since IL-10 is a secreted protein its presence in a certain layer does not necessarily mean that it was expressed there. 

In IL-10 KO mice (Figure 8), the location profile of the secreted IL-10 was similar to that obtained in wild type mice, being present in the epithelium and in the endothelium, although in general, low intensity of fluorescence was observed. HA-SLN_EE_ also showed the highest intensity of fluorescence in IL-10 KO mice, although the differences among formulations were less marked than in wild type mice. It should be considered that the capacity of the cells to be transfected and to produce the transgene protein could be affected by the altered condition derived of the deficiency in IL-10. In fact, the histological structure of the corneas showed differences between IL-10 KO and wild type mice, with the presence of gaps in the former (Figure 9). In this sense, the disorganization of the corneal layers in IL-10 KO mice probably results in a reduced barrier for diffusion of the secreted protein. 

## 5. Conclusions

Topical administration of eye drops containing SLN-based gene delivery systems have shown to be a feasible strategy to address corneal inflammation by de novo IL-10 production. The formulation of SLN-based vectors with PVA as viscosity modifier provided the system with the adequate versatility needed to overcome all the extra and intracellular barriers for a successful transfection. After 3 days of treatment by topical instillation, the multi-component nanosystem mainly transfected corneal epithelial cells in both wild type and IL-10 KO mice, HA-formulations combined with PVA being the most effective ones. IL-10 was even capable of reaching the endothelial layer. These promising results highlight the possible contribution of non-viral gene augmentation therapy to the future clinical approach of corneal gene therapy, although additional studies are necessary to improve and follow up long term expression of IL-10 and its impact on corneal inflammation.

## Figures and Tables

**Table 1:** Weight ratios of the complexes.

Name of the Complex	Weight Ratio
DX-SLN_EE_	DX:P:DNA:SLN_EE_ 1:2:1:5
HA-SLN_EE_	HA:P:DNA:SLN_EE_ 0.5:2:1:2
DNA-SLN_C_	DNA:SLN_C_ 1:10
HA-SLN_C_	HA:P:DNA:SLN_C_ 0.5:1:1:10

DX: dextran; HA: hyaluronic acid; P: protamine; SLN: solid lipid nanoparticle.

**Table 2:** Physical characterization of nanoparticles and SLN_EE_- and SLN_C_-based vectors bearing the plasmid pcDNA3-EGFP or the plasmid pUNO1-hIL10.

	Size (nm)	PDI	Zeta Potential (mV)
**SLN_EE_**	202.2 ± 28.2 *	0.25 ± 0.01	+51.3 ± 2.2
**HA-SLN_EE_**			
**pcDNA3-EGFP**	204.9 ± 15.0	0.18 ± 0.07	+29.2 ± 3.1
**pUNO1-hIL10**	266.1 ± 11.4	0.34 ± 0.03	+29.7 ± 1.2
**DX-SLN_EE_**			
**pcDNA3-EGFP**	177.3 ± 23.2 ^#^	0.33 ± 0.06	+39.3 ± 1.5 ^#^
**pUNO1-hIL10**	159.4 ± 4.9 *^‡^*	0.27 ± 0.02	+34.1 ± 0.6 *^‡^*
**SLN_C_**	453.9 ± 13.6	0.26 ± 0.03	+33.8 ± 2.5
**DNA-SLN_C_**			
**pcDNA3-EGFP**	404.1 ± 7.2 ^#^	0.27 ± 0.02	+20.8 ± 1.5
**pUNO1-hIL10**	447.9 ± 17.0 *^‡^*	0.29 ± 0.04	+14.7 ± 0.7 ^&^
**HA-SLN_C_**			
**pcDNA3-EGFP**	368.5 ± 7.4	0.24 ± 0.02	+21.9 ± 0.9
**pUNO1-hIL10**	374.7 ± 14.5	0.29 ± 0.02	+15.8 ± 2.4 ^&^

* *p* < 0.05 with respect to SLN_C_; ^#^ *p* < 0.05 with respect to the other vectors bearing the plasmid pcDNA3-EGFP; *^‡^ p* < 0.05 with respect to the other vectors bearing the plasmid pUNO1-hIL10; ^&^ *p* < 0.05 with respect to the same vector bearing the plasmid pcDNA3-EGFP. PDI: polydispersity index; DX: dextran; HA: hyaluronic acid; SLN: solid lipid nanoparticle. *n* = 3; data are expressed as mean ± standard deviation.

**Table 3:** High coefficient of determination (R^2^), viscosity (mPa·s) at shear rate of 10 and 500 s^−1^, consistency coefficient (k; Pa·s^n^) and flow behavior index (n) values of vectors and plasmid pcDNA3-EGFP solution with and without PVA.

Sample	R^2^	Viscosity 10 s^−1^ (mPa·s)	Viscosity 500 s^−1^ (mPa·s)	K (Pa·s^n^)	*n*
Water	0.9947	0.73	0.87	0.001	1.065
Plasmid solution	0.9847	0.78	0.98	0.001	1.11
Plasmid solution + PVA	0.9995	2.14	1.91	0.002	0.976
HA-SLN_EE_	0.9588	18.40	2.92	0.067	0.519
HA-SLN_EE_ + PVA	0.9592	8.26	2.38	0.015	0.681
DX-SLN_EE_	0.9921	1.60	1.00	0.002	0.876
DX-SLN_EE_ + PVA	0.9969	3.31	2.16	0.004	0.879
HA-SLN_C_	0.8642	10.70	1.75	0.028	0.525
HA-SLN_C_ + PVA	0.9995	3.29	3.08	0.003	0.989

PVA: polyvinyl alcohol; HA: hyaluronic acid; DX: dextran; SLN: solid lipid nanoparticle.

**Table 4:** pH measurements of plasmid solutions and vectors with and without PVA.

Sample	pH
Plasmid solution	7.4 ± 0.13
Plasmid solution + PVA	7.3 ± 0.13
HA-SLN_EE_	7.3 ± 0.03
HA-SLN_EE_ + PVA	7.2 ± 0.01
DX-SLN_EE_	7.5 ± 0.10
DX-SLN_EE_ + PVA	7.0 ± 0.18
HA-SLN_C_	4.0 ± 0.07
HA-SLN_C_ + PVA	4.3 ± 0.11

PVA: polyvinyl alcohol; HA: hyaluronic acid; DX: dextran; SLN: solid lipid nanoparticle. *n* = 3; data are expressed as mean ± standard deviation.

## Supplementary Materials

The following are available online at https://www.mdpi.com/1999-4923/12/6/584/s1, Figure S1: Image of SLN_C_ acquired by transmission electronic microscopy TEM. Figure S2: Capacity of the vectors to bind, protect, and release the pcDNA3-EGFP plasmid (A) and the pUNO1-hIL10 plasmid (B). Table S1: High coefficient of determination (R2), viscosity at shear rate of 10 and 500 s^−1^ (mPa s), consistency coefficient (k), and flow behavior index (n) values of vectors and plasmid solutions with and without PVA. Figure S3: Viscosity curve at shear rates from 5 to 1000 s^−1^ of water, the solution of plasmid pUNO1-hIL10 solution and vectors, with and without PVA. References [11,34,71] are cited in the supplementary materials.
