# Supplementary material for: Topical Administration of SLN-Based Gene Therapy for the Treatment of Corneal Inflammation by De Novo IL-10 Production
Source: Pharmaceutics. 2020 Jun 23;12(6):584. doi: 10.3390/pharmaceutics12060584 (PMC7355708; doi:10.3390/pharmaceutics12060584)
Supplement: Supplementary file 1 [file pharmaceutics-12-00584-s001.pdf]

pharmaceutics

Supplementary Materials: Topical Administration of SLN-Based Gene Therapy for the Treatment of Corneal Inflammation by De Novo IL-10 Production

Mónica Vicente-Pascual, Itziar Gómez-Aguado, Julen Rodríguez-Castejón, Alicia Rodríguez-Gascón, Elisabetta Muntoni, Luigi Battaglia, Ana del Pozo-Rodríguez * and María Ángeles Solinís Aspiazu *

1. Transmission electronic microscopy (TEM) images

Visualization of SLN_C was performed using electron microscopy negative staining. For that purpose, 10 µl of the sample was adhered onto glow discharged carbon coated grids for 60 s. Then the remaining liquid was removed by blotting on filter paper, and the samples were stained with 2% uranyl acetate for 60 s. SLN_C were visualized using a Philips EM208S TEM and digital images were acquired on an Olympus SIS purple digital camera. Technical and human support for TEM was provided by the General Service (SGIker) of Analytical Microscopy and High Resolution in Biomedicine at the University of the Basque Country UPV/EHU. TEM images of the SLN_{EE} were previously published [1].

TEM photograph of the SLN_C (Figure S1) showed the spherical shape of the nanoparticles.

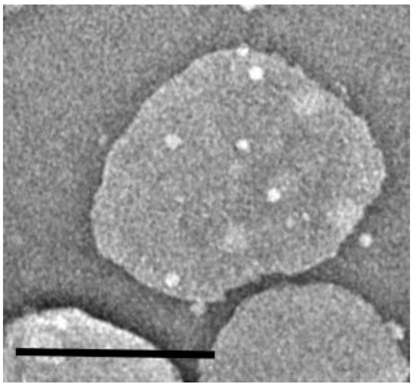

Figure S1. Image of SLN_C acquired by transmission electronic microscopy TEM. Scale bar: 500 nm. SLN: Solid lipid nanoparticle.

2. Ability of the vectors to bind, protect and release the plasmid DNA

In order to evaluate DNA binding efficacy of the SLN_C, as well as their protection and release capacity, a 0.7% agarose gel electrophoresis containing Gel Red™ was employed (Figure S2). Assessment of the ability of the vectors to bind electrostatically pcDNA3-EGFP and pUNO1-hIL10 plasmids was performed by preparing the complexes at a final concentration of 0.03 µg/µl of DNA in MilliQ™ water. This concentration of DNA was also subjected to 1 U DNase I/2.5 µg DNA during 30 minutes at 37ºC to study the protection capacity of the complexes. Finally, the release of DNA from the vectors was performed with a SDS solution (4%) to a final concentration of 1%. As control, naked pcDNA3-EGFP or pUNO1-hIL10 plasmid and 1 kb DNA ladder from NIPPON Genetics Europe (Dueren, Germany) were added. For the analysis of the gel, an Uvitec Uvidoc D-55-LCD-20M Auto transilluminator was used as previously reported [34]. DX-SLN_{EE} and HA-SLN_{EE} were assessed in previous studies [2,3].

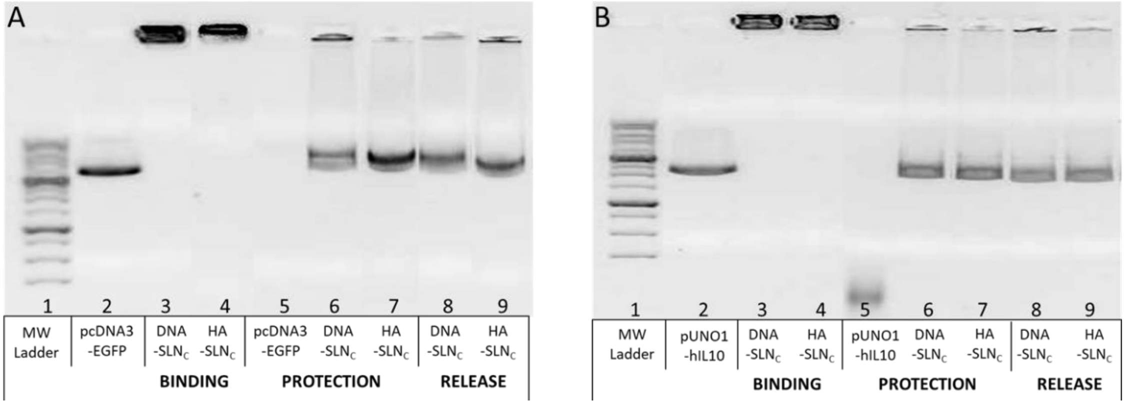

Figure S2. Capacity of the vectors to bind, protect and release the pcDNA3-EGFP plasmid (A) and the pUNO1-hIL10 plasmid (B). To study the release of the plasmid, the samples were treated with SDS, and for the protection assay, the samples were mixed first with DNase I and later with SDS to release the plasmid DNA. MW: molecular weight; SDS: sodium dodecyl sulphate; DNase I: deoxyribonuclease I.

As can be seen in lanes 3 and 4, DNA was completely bound to the vectors, since no band was observed on the correspondent lanes, and the plasmid was detected on the loading wells.

The release of the plasmid was evaluated after treating the complexes with SDS for 5 minutes. The DNA bands in lanes 8 and 9 indicate that both vectors were able to release the plasmids, although not completely, since the plasmids were also partially detected in the loading wells, especially in the case of the vectors prepared with the IL-10 plasmid (Figure S2B).

3. Rheology results with the plasmid pUNO1-hIL10

Table S1 and Figure S3 summarizes the results obtained in the rheological studies with the plasmid pUNO1-hIL10 and the vectors prepared with it. The results are consistent with those obtained with the plasmid pcDNA3-EGFP.

Table S1. High coefficient of determination (R^2), viscosity (mPa·s) at shear rate of 10 and 500 s^{-1}, consistency coefficient (k; $Pa·s^n$) and flow behaviour index (n) values of vectors and plasmid solutions with and without PVA.

	R^2	Viscosity 10 s^{-1} (mPa·s)	Viscosity 500 s^{-1} (mPa·s)	K ($Pa·s^n$)	n
Water	0.9921	0.75	0.90	0.001	1.070
Plasmid solution	0.9924	1.21	0.93	0.001	1.053
Plasmid solution + PVA	0.9960	2.05	1.49	0.002	0.954
HA-SLN$_{EE}$	0.9697	22.41	2.77	0.086	0.465
HA-SLN$_{EE}$ + PVA	0.9980	6.26	2.36	0.012	0.742
DX-SLN$_{EE}$	0.9981	1.73	1.08	0.002	0.878
DX-SLN$_{EE}$ + PVA	0.9952	4.00	2.82	0.006	0.883
HA-SLN$_{C}$	0.9550	12.29	1.64	0.029	0.523
HA-SLN$_{C}$ + PVA	0.9987	4.59	3.04	0.006	0.909

PVA: polyvinyl alcohol; HA: hyaluronic acid; DX: dextran; SLN: solid lipid nanoparticle.

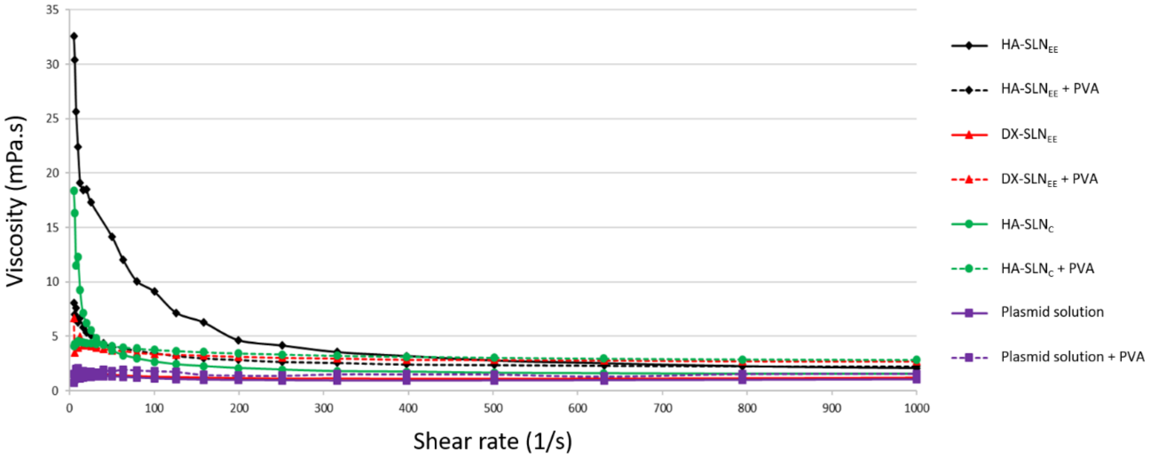

Figure S3. Viscosity curve at shear rates from 5 to 1000 s^{-1} of water, the solution of plasmid pUNO1-hIL10 solution and vectors, with and without PVA.

PVA: polyvinyl alcohol; HA: hyaluronic acid; DX: dextran; SLN: solid lipid nanoparticle

The plasmid solutions, with and without PVA, and HA-SLN$_C$ with PVA showed a flow behaviour similar to water (n index near 1), indicating Newtonian behaviour, whereas the values of n were lower in all the SLN$_{EE}$-based vectors, with and without PVA, and the vector HA-SLN$_C$ without PVA This is indicative of a pseudoplastic behaviour.

References

1. Apaolaza, P.S.; del Pozo-Rodríguez, A.; Torrecilla, J.; Rodríguez-Gascón, A.; Rodríguez, J.M.; Friedrich, U.; Weber, B.H.F.; Solinís, M.A. Solid lipid nanoparticle-based vectors intended for the treatment of X-linked juvenile retinoschisis by gene therapy: In vivo approaches in Rs1h-deficient mouse model. *J. Control. Release* **2015**, *217*, 273–283, doi:10.1016/j.jconrel.2015.09.033.
2. Vicente-Pascual, M.; Albano, A.; Solinís, M.Á.; Serpe, L.; Rodríguez-Gascón, A.; Foglietta, F.; Muntoni, E.; Torrecilla, J.; del Pozo-Rodríguez, AD.; Battaglia, L. Gene delivery in the cornea: In vitro & ex vivo evaluation of solid lipid nanoparticle-based vectors. *Nanomed. J.* **2018**, *13*, 1847–1864, doi:10.2217/nnm-2018-0112.
3. Delgado, D.; del Pozo-Rodríguez, A.; Solinís, M.Á.; Avilés-Triqueros, M.; Weber, B.H.F.; Fernández, E.; Rodríguez-Gascón, A. Dextran and Protamine-Based Solid Lipid Nanoparticles as Potential Vectors for the Treatment of X-Linked Juvenile Retinoschisis. *Hum. Gene Ther.* **2012**, *23*, 345–355, doi:10.1089/hum.2011.115.
